# Using the World Health Organization Disability Assessment Schedule 2.0 to assess disability in veterans with posttraumatic stress disorder

**DOI:** 10.1371/journal.pone.0220806

**Published:** 2019-08-07

**Authors:** Michelle J. Bovin, Eric C. Meyer, Nathan A. Kimbrel, Sarah E. Kleiman, Jonathan D. Green, Sandra B. Morissette, Brian P. Marx

**Affiliations:** 1 National Center for PTSD at VA Boston Healthcare System, Boston, Massachusetts, United States of America; 2 Boston University School of Medicine, Boston, Massachusetts, United States of America; 3 Department of Veterans Affairs VISN 17 Center of Excellence for Research on Returning War Veterans, Waco, Texas, United States of America; 4 Central Texas Veterans Healthcare System, Waco, Texas, United States of America; 5 Texas A&M University Health Science Center, College of Medicine, College Station, Texas, United States of America; 6 Durham Veterans Affairs Medical Center, Durham, North Carolina, United States of America; 7 VA Mid-Atlantic Mental Illness Research, Education, and Clinical Center, Durham, North Carolina, United States of America; 8 Department of Psychiatry and Behavioral Sciences, Duke University Medical Center, Durham, North Carolina, United States of America; 9 Department of Psychology, The University of Texas at San Antonio, San Antonio, Texas, United States of America; Stellenbosch University, SOUTH AFRICA

## Abstract

The introduction of the *Diagnostic and Statistical Manual of Mental Disorders (DSM-5)* was accompanied by the elimination of the Global Assessment of Functioning (GAF) scale, which was previously used to assess functioning. Although the World Health Organization Disability Assessment Schedule, Version 2.0 (WHODAS 2.0) was offered as a measure for further study, widespread adoption of the WHODAS 2.0 has yet to occur. The lack of a standardized instrument for assessing posttraumatic stress disorder (PTSD)-related disability has important implications for disability compensation. Accordingly, this study was designed to determine and codify the utility of the WHODAS 2.0 for assessing PTSD-related disability. Veterans from several VA medical centers (N = 1109) were included. We examined PTSD using several definitions and modalities and considered results by gender and age. Across definitions and modalities, veterans with PTSD reported significantly greater WHODAS 2.0 total (large effects; all *t*s > 6.00; all *p*s < .01; all Cohen’s *d*s > 1.03) and subscale (medium-to-large effects; all *t*s > 2.29; all *p*s < .05; all Cohen’s *d*s > .39) scores than those without PTSD. WHODAS 2.0 scores did not vary by gender; however, younger veterans reported less disability than older veterans (small effects; all *F*s > 4.30; all *p*s < .05; all η^2^s < .05). We identified 32 as the optimally efficient cutoff score for discriminating veterans with and without PTSD-related disability, although this varied somewhat by age and gender. Findings support the utility of the WHODAS 2.0 in assessing PTSD-related disability.

## Introduction

The introduction of the fifth edition of the *Diagnostic and Statistical Manual of Mental Disorders (DSM-5)* [1] was accompanied by the elimination of the Global Assessment of Functioning (GAF) scale, a clinician rating of global psychological, social, and occupational functioning. Although the *DSM-5* Task Force recommended the World Health Organization Disability Assessment Schedule, Version 2.0 (WHODAS 2.0) [2] as a replacement for the GAF, the American Psychiatric Association (APA) Board of Trustees did not approve the WHODAS 2.0 for this purpose, citing a need for additional data supporting its utility in improving patient care [3]. To date, few studies have responded to the APA’s call for additional research on the WHODAS 2.0 (e.g., [4–6]), and only one has examined it in a sample of individuals with posttraumatic stress disorder [7].

Having a standardized metric to assess mental health-related disability is particularly important for PTSD, as it is one of the most common conditions for which people receive disability compensation [8–10]. Use of a standardized tool is essential for ensuring that decisions are both accurate and consistent. Indeed, a multi-site randomized trial found that expert reviewers rated disability assessments that used standardized tools for assessing functional impairment (i.e., WHODAS 2.0) as being significantly more complete than those conducted using non-standardized assessment instruments [11].

Despite the importance of having a standardized measure of PTSD-related disability, widespread adoption of the WHODAS 2.0 for this purpose has not occurred. In many ways, the WHODAS 2.0—which provides a global disability score, six domain scores, and demonstrates strong psychometric properties [2]—is ideally suited for this role. Importantly, the WHODAS 2.0 improves upon the GAF in that it does not confound symptom severity with functional impairment [12]. However, there is currently no established threshold for interpreting WHODAS 2.0 scores in relation to the PTSD criterion of clinically significant impairment. Although Marx and his colleagues [7] established an initial threshold, it was computed in a small, disability-seeking sample using the WHODAS 2.0 interview version. Therefore, although their work provides an initial starting point, they recommended replication in a larger study to ensure generalizability. Further, there is concern that no single cutoff score would be meaningful across all demographic categories [13]; this question has not yet been examined empirically. Addressing these concerns is paramount for establishing the clinical utility of the self-report version of the WHODAS 2.0, and the subsequent adoption of this instrument for the assessment of PTSD-related disability.

In this study, we address these concerns. Specifically, in a large sample of veterans, we explored the scope of the WHODAS 2.0’s clinical utility by examining whether people with PTSD reported significantly greater disability than those without PTSD, regardless of the *DSM* criteria (the fourth edition of the *DSM* [*DSM-IV*] versus *DSM-5*) or assessment modality (interview versus self-report) used. These are important issues, as many disability-related decisions were established based on *DSM-IV* criteria, and both self-report and interview-based assessments of PTSD are commonly used both within clinical and disability assessment settings. We then examined the effect of age and gender on WHODAS 2.0 scores, in response to Konecky and colleagues’ [13] concern that the utility of the WHODAS 2.0 might be undercut by varying reliability across demographic groups. Finally, we conducted signal detection analyses to identify an overall cutoff score for the WHODAS 2.0, as well as cutoff scores by gender and age.

## Method

### Participants

Participants included 1109 veterans from four independent samples (see Table 1 for demographic characteristics). Sample 1 included veterans recruited through the Boston VA Healthcare System for a study designed to validate the PTSD Checklist for *DSM-5* [14] and the Clinician Administered PTSD Scale for *DSM-5* [15]. Inclusion criteria included veteran status, aged 18 or older, literacy in English, and endorsement of at least one traumatic event and at least one PTSD symptom. As reported elsewhere [16] 142 participants completed the study. Data from six participants were dropped from current analyses because they did not complete the WHODAS 2.0, leaving a total sample of 136 participants.

**Table 1:** Sample characteristics.

Characteristic	Sample 1 (*n* = 136)	Sample 2 (*n* = 275)	Sample 3 (*n* = 390)	Sample 4 (*n* = 308)	Total Sample (N = 1,109)
Age (*M*, *SD*)	53.80 (11.89)	51.77 (11.79)	51.60 (11.13)	38.74 (9.75)	48.31 (12.54)
Gender (% Female)	10.3	11.7	13.9	32.5	18.1
Race (%)					
Caucasian	65.9	68.0	59.0	58.7	62.3
Black	28.9	25.5	24.7	34.0	28.0
Asian	0	0.4	6.2	2.0	2.8
Pacific Islander	0	0	0	1.7	0.4
Native American	0	2.2	2.7	5.7	3.2
Ethnicity (%)					
Hispanic/Latino	5.1	2.9	7.2	19.5	9.3
Married (%)	24.6	21.7	18.0	64.2	32.4
Years of Education (*M*, *SD*)	13.82 (2.29)	13.53 (2.38)	13.46 (2.57)	14.15 (2.12)	13.72 (2.38)
Combat Exposure (%)	38.1	31.2	31.5	90.6	46.0
Military Conflict (%)					
Vietnam	31.5	32.0	21.8	1.3	18.5
Iraq/Afghanistan	20.0	18.2	15.4	100	41.0
First Gulf War	7.7	14.6	11.6	18.2	13.1
Korea	4.6	2.4	3.2	0	2.2
Bosnia	2.3	0.8	1.6	14.0	4.9
World War II	0	2.0	0.6	0	0.9
Other	16.9	14.6	18.3	18.8	16.6
Did Not Deploy	23.1	16.6	30.4	N/A	16.6
WHODAS 2.0					
Total Score *M (SD*)	43.82 (23.04)	41.75 (24.19)	39.79 (26.38)	30.46 (23.94)	38.18 (25.24)
Cognition *M (SD*)	7.74 (4.86)	7.40 (4.90)	7.26 (5.16)	5.86 (5.13)	6.97 (5.10)
Mobility *M (SD*)	7.24 (5.00)	6.70 (5.08)	6.56 (5.19)	4.73 (4.69)	6.17 (5.08)
Self-Care *M (SD*)	2.17 (2.53)	2.23 (2.86)	2.32 (3.07)	1.13 (2.16)	1.95 (2.76)
Getting Along *M (SD*)	6.61 (4.87)	6.77 (4.68)	6.10 (4.95)	5.13 (4.98)	6.06 (4.92)
Life Activities *M (SD*)	7.88 (6.52)	6.71 (5.77)	6.69 (6.83)	6.59 (6.28)	6.81 (6.39)
Participation *M (SD*)	12.18 (6.04)	11.97 (6.83)	11.41 (7.52)	7.48 (6.95)	10.60 (7.27)
Met Criteria for PTSD (%)					
PCL-C	70.8%	–	58.1%	39.7%	53.2%
PCL-5	66.9%	–	55.3%	–	58.6%
CAPS-IV	–	26.7%	–	35.6%	31.2%
CAPS-5	53.4%	–	–	–	53.4%

*Note*. WHODAS 2.0 = World Health Organization Disability Assessment Schedule, Version 2.0. PCL-C = PTSD Checklist, Civilian Version; PCL-5 = PTSD Checklist for *DSM-5*; CAPS-IV = Clinician Administered PTSD Scale for *DSM-IV*; CAPS-5 = Clinician Administered PTSD Scale for *DSM-5*.

Sample 2 included veterans recruited through the Boston VA Healthcare System for one phase of a study to develop and validate the Inventory of Psychosocial Functioning (IPF) [17]. Included participants were aged 18 or older and were literate in English. Of the 285 participants who completed this phase of the larger study, data from 10 were dropped from current analyses because they had not completed the WHODAS 2.0. This left a total of 275 participants.

Sample 3 included veterans recruited from both the Boston VA Healthcare System and the VA Pacific Islands Healthcare System; these veterans were recruited for another phase of a study to develop and validate the IPF [17].There was no overlap among participants in Samples 2 and 3. Like Sample 2, inclusion criteria included veteran status, being aged 18 or older, and literacy in English. Of the 394 participants who completed this phase of the larger study, four were dropped from current analyses due to missing data on the WHODAS 2.0. This left a final sample of 390 participants.

Sample 4 included veterans recruited from the Central Texas Veterans Healthcare System for a study examining predictors of post-deployment mental health and functional outcomes. Inclusion criteria included veteran status, having deployed in support of the post-9/11 wars in Iraq and Afghanistan, and absence of a bipolar and/or psychotic disorder diagnosis. In addition, although receiving treatment was not a condition of eligibility, those who were receiving treatment were required to be stable in treatment to ensure that symptoms and impairment were not due to recently starting or stopping treatment. Recruitment involved over-sampling for women veterans and those with probable PTSD. Of the 309 veterans who completed the study, one was excluded because of missing data on the WHODAS 2.0. This left a final sample of 308 participants.

### Measures

#### WHODAS 2.0

The WHODAS 2.0 [2] is a 36-item self-report measure that assesses disability related to health conditions in the past 30 days. The measure assesses disability across six domains: cognition, mobility, self-care, getting along with others, life activities, and participation. Participants respond to each item on a 5-point scale from 0 (*No Difficulty*) to 4 (*Extreme Difficulty/Cannot Do*). In the current study, we used the simple scoring method, in which items are summed to total scores for each domain, with higher scores indicating greater disability. A global functional disability score was calculated by summing all 36 items. The WHODAS 2.0 has demonstrated excellent reliability and validity [2, 18]. Across study samples, Cronbach’s α for the overall score of global functional disability was .97, with the domain scale scores ranging from .83 (self-care) to .94 (life activities).

#### Clinician administered PTSD scale for *DSM-IV* (CAPS-IV)

The CAPS-IV [19] is a structured diagnostic interview that assesses *DSM-IV* PTSD symptom severity and diagnosis. For each symptom, a clinician rates two dimensions, frequency and intensity, on separate 5-point scales. The CAPS-IV consistently demonstrates excellent psychometric properties [20]. In the current study, the CAPS-IV was used to assess *DSM-IV* PTSD symptoms and PTSD diagnosis in Samples 2 and 4. In Sample 2, the CAPS-IV was administered by doctoral-level clinicians who participated in regular reliability meetings. Interrater reliability was excellent (κ = .78). For Sample 4, interviews were conducted by masters- and doctoral-level clinicians, and weekly diagnostic review meetings supervised by doctoral-level staff were held to reach diagnostic consensus. The Frequency ≥ 1/Intensity ≥ 2 (F1/I2) rule was used to calculate *DSM-IV* PTSD diagnostic status. According to this rule, a PTSD symptom is present if the Frequency score is ≥ 1 and the Intensity score is ≥ 2; a PTSD diagnosis is derived by first dichotomizing the items, and then following the *DSM-IV* algorithm for PTSD (one reexperiencing symptom, three avoidance and numbing symptoms, and two hyperarousal symptoms) [21]. In addition, two of the items measuring social and occupational impairment were used to create an index of interviewer-rated PTSD-related psychosocial functioning. Participants with scores of 2 or above on either item were coded as having PTSD-related impairment. In the current study, of the 465 participants who completed the CAPS-IV, 31.2% (*n* = 145) met criteria for the PTSD diagnosis, and 57.4% (*n* = 299) met criteria for PTSD-related psychosocial impairment.

#### CAPS-5

The CAPS-5 [15] is a structured diagnostic interview that assesses *DSM-5* PTSD symptom severity and diagnosis. Clinicians rate each symptom on a 5-point severity scale, ranging from 0 (*Absent*) to 4 (*Extreme/incapacitating*). Initial examination of the CAPS-5 suggests that it retains the same strong psychometric properties as the CAPS-IV [22]. In the current study, the CAPS-5 was used to assess *DSM-5* PTSD symptoms and PTSD diagnosis in Sample 1. The CAPS-5 was administered by masters- and doctoral-level clinicians who participated in regular reliability meetings. Interrater reliability was excellent (κ = .78). We used the CAPS-5 to calculate PTSD diagnostic status according to *DSM-5* using the SEV2/26 rule [22]. According to this rule, a PTSD symptom is present if the Severity score is ≥ 2; a PTSD diagnosis is derived by first dichotomizing the items, and then following the *DSM-5* algorithm for PTSD (one intrusion symptom, one avoidance symptom, two negative alteration in cognition or mood symptoms, and two arousal and reactivity symptoms). In addition, we used the CAPS-5 to create an index of interview-rated, PTSD-related psychosocial functional impairment. Participants with a score of 2 or above on the item assessing social functional impairment and/or the item assessing occupational functional impairment were coded as having PTSD-related impairment. In the current study, of the 131 participants who completed the CAPS-5, 53.4% (*n* = 70) met criteria for the PTSD diagnosis, and 68.9% (*n* = 93) met criteria for PTSD-related psychosocial impairment.

#### PTSD checklist-civilian version (PCL-C)

The PCL-C [23] is a 17-item self-report measure designed to assess symptoms of PTSD according to *DSM-IV*. Respondents rate each item on a 5-point scale ranging from 1 (*Not at all*) to 5 (*Extremely*). A PCL-C total score is then calculated by summing each of the 17 items, with higher scores indicating higher levels of PTSD symptom severity. PCL-C scores consistently demonstrate strong psychometric properties [24]. In the current study, the PCL-C was completed by veterans in Samples 1, 3, and 4. Cronbach’s α was .96 for the combined samples. We used the PCL-C to determine probable PTSD diagnostic status according to *DSM-IV* using a cutoff score of 44 [25]. In the current study, of the 786 participants who completed the PCL-C, 53.2% (*n* = 418) met criteria for probable PTSD.

#### PCL-5

The PCL-5 [14] is a 20-item self-report measure designed to assess PTSD symptoms according to *DSM-5*. Respondents rate each item on a 5-point scale ranging from 0 (*Not at all*) to 4 (*Extremely*). PCL-5 total scores are calculated by summing the 20 items, with higher scores indicating higher levels of PTSD symptom severity. Like the PCL-C, the PCL-5 has demonstrated excellent psychometric properties [16]. In the current study, Samples 1 and 3 completed the PCL-5. Cronbach’s α was .96 for the combined samples. We used the PCL-5 to determine probable PTSD diagnostic status according to *DSM-5* using a cutoff score of 33 [16]. In the current study, of the 473 participants who completed the PCL-5, 58.6% (*n* = 277) met criteria for probable PTSD.

#### Demographics

All participants provided information on their age, sex, education, ethnicity, race, marital status, and military history via self-report.

### Procedure

As reported elsewhere [16] participants in Sample 1 were recruited from the Boston VA Healthcare System. All participants were listed in a large database of veterans who had previously consented to be contacted regarding research participation. Participants completed a battery of self-report questionnaires and, upon completion, were assessed with a clinical interview. For each participant, the WHODAS 2.0 was administered fifth (of 12 self-report measures), the PCL-C was administered seventh, and the PCL-5 was administered eighth. At study completion, participants were debriefed and compensated monetarily. Consistent with APA ethical standards, IRB approval was obtained from the VA Boston Healthcare System IRB for data collection, and all participants provided written informed consent prior to participation.

Participants in Sample 2 were recruited from a VA hospital using both fliers and from a large database of veterans who had previously indicated that they would be interested in participating in research. Participants first completed a battery of self-report questionnaires and then participated in several diagnostic interviews. For each participant, the WHODAS 2.0 was administered fifth (of 11 self-report measures). At the end of participation, veterans were debriefed and compensated for their time [17]. Consistent with APA ethical standards, IRB approval was obtained from the VA Boston Healthcare System IRB for data collection, and all participants provided written informed consent prior to participation.

Participants in Sample 3 were recruited from two VA hospitals from both fliers and from the research database discussed above. Participants completed a self-report questionnaire battery. For each participant, the WHODAS 2.0 was administered sixth (of 23 self-report measures), the PCL-C was administered tenth, and the PCL-5 was administered eleventh. After participation, they were debriefed and compensated [17]. Consistent with APA ethical standards, IRB approval was obtained from both the VA Boston Healthcare System IRB and the VA Pacific Islands Healthcare System IRB for data collection, and all participants provided written informed consent prior to participation.

Sample 4 participants were recruited through posted announcements and advertisements at a VA medical center, as well as through direct mailings to oversample for female veterans and veterans with mental health diagnoses. Participants completed clinical interviews and then were administered a battery of self-report measures. Five separate packets of the 30 self-report measures were randomized across participants, with each varying the order of measure presentation. The placement of the WHODAS 2.0 ranged from tenth to twenty sixth, whereas the placement of the PCL-C ranged from fifth to twenty third. Therefore, in some cases the WHODAS 2.0 was administered prior to the PCL-C; in other cases, this order was reversed. Upon completion of the study, all participants were compensated for participation. Consistent with APA ethical standards, IRB approval was obtained from the Central Texas VA Healthcare System IRB for data collection, and all participants provided written informed consent prior to participation.

### Data analysis plan

Except for signal detection analyses, all analyses were conducted using SPSS version 25. First, we calculated means and standard deviations for the WHODAS 2.0 total score and each of the six subscales scores. Next, we conducted *t*-tests to determine if participants with and without PTSD demonstrated significantly different WHODAS 2.0 scores. We also conducted *t*-tests and ANOVAs to determine whether WHODAS 2.0 scores differed as a function of gender and age. Age was examined as a categorical variable that represented three levels: younger veterans (aged 18–-34), mid-aged veterans (aged 35–59), and older veterans (aged 60 and older). This categorization of age is consistent with other studies that have examined the relation between age and functioning; these studies have found that some impairment differences may be evident beginning at approximately 35 years of age [26] and again at 60 years of age [27]. Effect sizes for *t*-tests were evaluated using Cohen’s *d*, such that small = 0.2, medium = 0.5, and large = 0.8, and effect sizes for ANOVAs were evaluated using η^2^, where small = 0.01, medium = 0.06, and large = 0.14 [28]. We used a Holm [29] correction for the *t*-tests, overall *F*-tests, and each set of post hoc tests to simultaneously maintain power and protect against Type I error given the seven omnibus tests (one for the WHODAS 2.0 total score and each of the six subscale scores), and for the ANOVAs, three post hoc tests, conducted in each set of analyses.

Signal detection analyses were conducted to identify cutoff scores for the overall sample and for participant groups stratified by gender and age. We examined diagnostic accuracy for each cutoff score on the WHODAS 2.0 with weighted κ coefficients as measures of test quality, including quality of sensitivity (κ[1]), specificity (κ[0]), and efficiency (κ[.5]). Unlike commonly-reported measures of test performance (e.g., sensitivity, specificity, and efficiency), weighted κ coefficients are calibrated for chance agreement between test and diagnosis [30]. Guidelines developed for judging levels of clinical significance suggest that κ ≤ 0.40 is poor, ≥ 0.41 and < 0.60 is fair, ≥ 0.60 and < 0.75 is good, and ≥ 0.75 is excellent [31].

Our analyses guided us to identify the optimally efficient cutoff (i.e., the WHODAS 2.0 cutoff with the highest κ[.5]) for each group. We opted to examine optimally efficient cutoffs because they minimize diagnostic errors [30]. To do so, we first calculated receiver operating characteristic (ROC) curves using the WHODAS 2.0 as the test variable and the CAPS impairment index as the criterion variable, examined the sensitivity and specificity for each value, and identified 5–10 possible WHODAS 2.0 total cutoff scores that represented the best balance of sensitivity and specificity. Next, we dichotomized the WHODAS 2.0 on each of these potential cutoff scores such that all scores at or above the potential cutoff score were coded as “1” and all values below the potential cutoff score were coded as “0.” We then ran crosstabs with the dichotomized WHODAS 2.0 variable as the test variable and the CAPS impairment index as the criterion variable. Because no participants were administered both the CAPS-IV and the CAPS-5, we combined the impairment indices from the two measures into a single variable. This allowed us to conduct signal detection analyses with all participants who had complete data on either CAPS version simultaneously, and to determine a cutoff score that was agnostic to the version of the *DSM* utilized. Using DAG_STAT [32], we calculated measures of test performance and test quality for each of the cutoff scores.

## Results

As can be seen in Table 1, the mean WHODAS 2.0 total score across the four samples was 38.18 (*SD* = 25.24, range = 0 to 143). Across the four samples, mean WHODAS 2.0 subscale scores ranged from 1.95 (*SD* = 2.76; self-care) to 10.60 (*SD* = 7.27; participation). Further, regardless of the definition or the assessment instrument used, participants with PTSD reported significantly more functional disability than those without PTSD on the WHODAS 2.0 total scale and on each of the subscales, with medium to large effect sizes (all *t*s > 2.20, all *p*s < .05, all Cohen’s *d*s > .39; see Table 2).

**Table 2:** Comparison of WHODAS means as a factor of PTSD diagnostic status, gender, and age^a^.

WHODAS.20	Total		Cognition		Mobility		Self-Care		Getting Along		Life Activities		Participation	
	*M* (*SD*)	*t/F*	*M* (*SD*)	*t/F*	*M* (*SD*)	*t/F*	*M* (*SD*)	*t/F*	*M* (*SD*)	*t/F*	*M* (*SD*)	*t/F*	*M* (*SD*)	*t/F*
**PTSD**														
PCL-C		24.77**		20.94**		17.41**		13.15**		19.84**		13.02**		22.93**
PTSD (*n* = 418)	52.29 (22.34)		9.65 (4.59)		8.33 (4.78)		2.84 (3.07)		8.47 (4.72)		9.40 (6.90)		14.43 (6.55)	
No PTSD (*n* = 368)	18.82 (15.25)		3.51 (3.63)		3.05 (3.70)		0.61 (1.44)		2.76 (3.22)		3.93 (4.68)		5.10 (4.68)	
Cohen’s *d*	1.75		1.48		1.24		0.93		1.41		0.93		1.64	
PCL-5		17.48**		14.96**		10.47**		8.42**		15.88**		9.91**		16.74**
PTSD (*n* = 277)	53.65 (21.95)		9.75 (4.48)		8.57 (4.88)		3.03 (2.99)		8.55 (4.66)		9.22 (6.98)		15.30 (6.21)	
No PTSD (*n* = 196)	22.41 (16.89)		4.04 (3.79)		4.04 (4.28)		1.03 (2.09)		2.89 (2.97)		3.84 (4.64)		6.67 (4.98)	
Cohen’s *d*	1.60		1.38		1.00		0.78		1.45		0.91		1.53	
CAPS-IV		10.46**		10.22**		5.71**		4.81**		9.57**		6.57**		9.50**
PTSD (*n* = 145)	55.30 (23.01)		10.46 (4.67)		8.02 (4.93)		2.88 (3.29)		9.57 (4.68)		9.97 (6.68)		15.13 (6.52)	
No PTSD (*n* = 320)	32.06 (21.84)		5.80 (4.50)		5.26 (4.78)		1.43 (2.32)		5.31 (4.34)		5.79 (5.53)		8.75 (6.58)	
Cohen’s *d*	1.04		1.02		0.57		0.51		0.94		0.68		0.97	
CAPS-5		6.01**		4.41**		4.16**		2.30*		5.15**		3.20**		6.62**
PTSD (*n* = 70)	54.45 (20.39)		9.50 (4.58)		8.89 (4.72)		2.70 (2.62)		8.56 (4.75)		9.64 (6.84)		15.17 (4.83)	
No PTSD (*n* = 61)	32.84 (20.67)		5.98 (4.50)		5.43 (4.78)		1.69 (2.38)		4.52 (4.11)		6.10 (5.65)		9.11 (5.65)	
Cohen’s *d*	1.05		0.78		0.73		0.40		0.91		0.56		1.15	
**Gender**		-0.48		0.11		0.79		-0.53		-1.33		-1.96		0.21
Male (*n* = 905)	37.97 (24.72)		6.97 (5.11)		6.23 (5.02)		1.93 (2.72)		5.97 (4.87)		6.61 (6.19)		10.60 (7.07)	
Female (*n* = 200)	38.99 (27.49)		6.93 (5.04)		5.92 (5.37)		2.04 (2.99)		6.48 (5.10)		7.69 (7.19)		10.47 (8.08)	
Cohen’s *d*	0.05		0.01		0.05		-0.04		-0.10		-0.16		0.02	
**Age**		7.70**		1.53		24.15**		7.19**		4.32		0.89		10.31**
1) 18–34 years (*n* = 200)	32.19 (23.32)		6.55 (4.79)		3.97 (4.50)		1.29 (2.25)		5.37 (4.78)		6.73 (5.86)		8.58 (7.14)	
2) 35–59 years (*n* = 691)	40.08 (25.53)		7.18 (5.19)		6.60 (5.07)		2.09 (2.87)		6.41 (5.04)		6.99 (6.57)		11.24 (7.32)	
3) 60+ years (*n* = 199)	37.83 (25.36)		6.71 (5.03)		6.89 (5.11)		2.16 (2.82)		5.68 (4.55)		6.31 (6.16)		10.40 (6.96)	
LSD	1 < 2; 1 = 3; 2 = 3		1 = 2 = 3		1 < 2; 1 < 3; 2 = 3		1 < 2; 1 < 3; 2 = 3		1 = 2 = 3		1 = 2 = 3		1 < 2; 1 < 3; 2 = 3	
η^2^	.01		.00		.04		.01		.01		.02		.00	

*Note*. WHODAS 2.0 = World Health Organization Disability Assessment Schedule, Version 2.0; CAPS-5 = Clinician Administered PTSD Scale for *DSM-5*; CAPS-IV = Clinician Administered PTSD Scale for *DSM-IV*; PCL-5 = PTSD Checklist for *DSM-5*; PCL-C = PTSD Checklist, Civilian Version. *t*-tests were used to compare PTSD diagnostic status and genders; ANOVAs were used to compare age groups. For ANOVAs, LSD was used as the post hoc test. Comparisons are represented by number, where 1 = 18–34 years, 2 = 35–59 years, and 3 = 60 years and older. Higher scores indicate more impairment.

^a^Kolmogorov-Smirnov tests indicated that our variables were not normally distributed (all *p*s < .01). Results based on non-parametric tests (i.e., Mann-Whitney tests instead of *t*-tests; Kruskal-Wallis tests ANOVAs) produced identical results to those of parametric tests. Due to both robustness against normality violations and ease of interpretability, we report results of the *t*-tests and ANOVAs here.

* < .05

** < .01

### Association between WHODAS scores and both gender and age

Results indicated that men and women did not significantly differ on the WHODAS 2.0 total scale score or on any of the subscale scores (all *t*s < ǀ1.96ǀ; all *p*s > .05; see Table 2); however, significant differences were observed between the three age groups on the WHODAS 2.0 total scale score and on the mobility, self-care, and participation subscale scores (all *F*s > 7.10; all *p*s < .01), with younger veterans having less disability. Simple effects tests revealed that for all significant omnibus tests, younger participants were less disabled than older participants, albeit with small effect sizes (all η^2^s < .05; see Table 2).

### Signal detection analyses

We first determined the optimal cutoff scores for identifying PTSD-related disability among all participants who had been administered the CAPS overall (*n* = 656) and by gender. For the full sample, the Area Under the Curve (AUC) for the WHODAS 2.0 total score was 0.80 (SE = .02; 95% *C*.*I*.: 0.76–0.83), and the optimally efficient total score was 32 (κ[.5] = .47, SE = 0.04, 95% *C*.*I*.: 0.40–-0.53; see Table 3). For men (*n* = 517), the AUC for the WHODAS 2.0 was 0.81 (SE = .02; 95% *C*.*I*.: 0.77–0.84), and the optimally efficient total score was 32 (κ[.5] = .49, SE = 0.04, 95% *C*.*I*.: 0.41–0.56). For women (*n* = 138), the AUC for the WHODAS 2.0 was 0.79 (SE = .04; 95% *C*.*I*.: 0.71–0.87), and the optimally efficient score was 31–32 (the two cutoff scores had identical psychometric properties; κ[.5] = .38, SE = 0.08, 95% *C*.*I*.: 0.23–0.53).

**Table 3:** Diagnostic utility of WHODAS total score for predicting functional impairment on the CAPS (either IV or 5).

Total Sample (*n* = 656)								
Measure (cutoff)	Sens	Spec	PPV	NPV	Eff	κ[0]	κ[.5]	κ[1]
WHODAS (31)	.77	.69	.79	.67	.74	.47	.46	.45
**WHODAS (32)**	**.77**	**.70**	**.79**	**.67**	**.74**	**.49**	**.47**	**.45**
WHODAS (33)	.75	.72	.80	.66	.74	.49	.46	.43
WHODAS (34)	.73	.73	.80	.65	.73	.51	.46	.41
WHODAS (35)	.70	.74	.80	.63	.72	.50	.43	.37

*Note*. WHODAS 2.0 = World Health Organization Disability Assessment Schedule, Version 2.0; Sens = sensitivity; Spec = specificity; PPV = positive predictive value; NPV = negative predictive value; Eff = efficiency; κ[0] = optimal specificity; κ[.5] = optimal effiency; κ[1] = optimal sensitivity.

Next, we examined whether different WHODAS 2.0 cutoff scores were required for the three age groups. For younger veterans (*n* = 142), the AUC for WHODAS 2.0 was .82 (SE = .04; 95% *C*.*I*.: 0.76–0.89), and the optimally efficient total score was 32 (κ[.5] = .49, SE = 0.07, 95% *C*.*I*.: 0.35–0.62). For mid-aged veterans (*n* = 388), the AUC for WHODAS 2.0 was .80 (SE = .02; 95% *C*.*I*.: 0.76–0.85), and the optimally efficient total score was 31 (κ[.5] = .45, SE = 0.05, 95% *C*.*I*.: 0.36–0.54). For older veterans (*n* = 111), the AUC for WHODAS 2.0 was .76 (SE = .05; 95% *C*.*I*.: 0.67–0.85), and the optimally efficient total score was 32 (κ[.5] = .47, SE = 0.08, 95% *C*.*I*.: 0.31–0.64).

Finally, we examined whether different cutoff scores were indicated for different age groups within each gender. For younger men (*n* = 95), the AUC was .87 (SE = .04; 95% *C*.*I*.: 0.80–0.94), and the optimally efficient total score was 32 (κ[.5] = .59, SE = 0.08, 95% *C*.*I*.: 0.43–0.75). For mid-aged men (*n* = 303), the AUC for WHODAS 2.0 was .81 (SE = .02; 95% *C*.*I*.: 0.77–0.86), and the optimally efficient total score was 31 (κ[.5] = .47, SE = 0.05, 95% *C*.*I*.: 0.36–0.57). For older men (*n* = 105), the AUC for WHODAS 2.0 was .74 (SE = .05; 95% *C*.*I*.: 0.65–0.84), and the optimally efficient total score was 32 (κ[.5] = .45, SE = 0.09, 95% *C*.*I*.: 0.28–0.62; see Table 4).

**Table 4:** Diagnostic utility of WHODAS total score for predicting functional impairment on the CAPS (either IV or 5) among younger, mid-aged, and older men.

**Younger Men (*n* = 95)**								
**Measure (cutoff)**	**Sens**	**Spec**	**PPV**	**NPV**	**Eff**	**κ[0]**	**κ[.5]**	**κ[1]**
WHODAS (31)	.77	.83	.88	.67	.79	.69	.57	.48
**WHODAS (32)**	**.77**	**.86**	**.90**	**.68**	**.80**	**.73**	**.59**	**.50**
WHODAS (33)	.75	.86	.90	.67	.79	.73	.57	.47
WHODAS (34)	.73	.89	.92	.66	.79	.77	.58	.46
WHODAS (35)	.72	.89	.91	.65	.78	.77	.56	.44
**Mid-Aged Men (*n* = 303)**								
**Measure (cutoff)**	**Sens**	**Spec**	**PPV**	**NPV**	**Eff**	**κ[0]**	**κ[.5]**	**κ[1]**
**WHODAS (31)**	**.83**	**.63**	**.75**	**.73**	**.74**	**.41**	**.47**	**.53**
WHODAS (32)	.82	.64	.75	.72	.74	.42	.46	.51
WHODAS (33)	.80	.65	.76	.71	.74	.43	.46	.50
WHODAS (34)	.78	.67	.76	.70	.73	.44	.45	.47
WHODAS (35)	.76	.68	.76	.68	.73	.44	.44	.44
**Older Men (*n* = 105)**								
**Measure (cutoff)**	**Sens**	**Spec**	**PPV**	**NPV**	**Eff**	**κ[0]**	**κ[.5]**	**κ[1]**
WHODAS (31)	.76	.67	.71	.72	.71	.40	.43	.46
**WHODAS (32)**	**.74**	**.71**	**.73**	**.72**	**.72**	**.44**	**.45**	**.46**
WHODAS (33)	.72	.71	.72	.71	.71	.43	.43	.43
WHODAS (34)	.72	.71	.72	.71	.71	.43	.43	.43
WHODAS (35)	.67	.71	.71	.67	.69	.39	.37	.35

*Note*. WHODAS 2.0 = World Health Organization Disability Assessment Schedule, Version 2.0; Sens = sensitivity; Spec = specificity; PPV = positive predictive value; NPV = negative predictive value; Eff = efficiency; κ[0] = optimal specificity; κ[.5] = optimal effiency; κ[1] = optimal sensitivity.

Younger men = aged 18–34 years; mid-aged men = aged 35–59 years; older men = aged 60 years and older.

For younger women (*n* = 47), the AUC for WHODAS 2.0 was .73 (SE = .08; 95% *C*.*I*.: 0.58–0.88), and the optimally efficient total score was 28–34 (these cutoff scores demonstrated identical psychometric properties; κ[.5] = .27, SE = 0.12, 95% *C*.*I*.: 0.03–0.50). For mid-aged women (*n* = 85), the AUC for WHODAS 2.0 was .79 (SE = .05; 95% *C*.*I*.: 0.69–0.89), and the optimally efficient total score was 34 (κ[.5] = .38, SE = 0.10, 95% *C*.*I*.: 0.18–0.57). Due to the small number of older women in our sample (*n* = 5), we could not conduct a signal detection analysis (see Table 5).

**Table 5:** Diagnostic utility of WHODAS total score for predicting functional impairment on the CAPS (either IV or 5) among younger, mid-aged, and older women.

**Younger Women (*n* = 47)**								
**Measure (cutoff)**	**Sens**	**Spec**	**PPV**	**NPV**	**Eff**	**κ[0]**	**κ[.5]**	**κ[1]**
WHODAS (27)	.50	.71	.68	.54	.60	.29	.21	.16
**WHODAS (28–34)**	**.42**	**.86**	**.79**	**.55**	**.62**	**.52**	**.27**	**.18**
WHODAS (35)	.35	.86	.75	.51	.57	.44	.19	.12
**Mid-Aged Women (*n* = 85)**								
**Measure (cutoff)**	**Sens**	**Spec**	**PPV**	**NPV**	**Eff**	**κ[0]**	**κ[.5]**	**κ[1]**
WHODAS (31)	.76	.64	.86	.48	.73	.45	.36	.30
WHODAS (32)	.76	.64	.86	.48	.73	.45	.36	.30
WHODAS (33)	.73	.68	.87	.47	.72	.49	.36	.28
**WHODAS (34)**	**.71**	**.73**	**.88**	**.47**	**.72**	**.55**	**.38**	**.29**
WHODAS (35)	.67	.73	.88	.43	.68	.52	.32	.23
**Older Women (*n* = 5)**^**a**^								

*Note*. WHODAS 2.0 = World Health Organization Disability Assessment Schedule, Version 2.0; Sens = sensitivity; Spec = specificity; PPV = positive predictive value; NPV = negative predictive value; Eff = efficiency; κ[0] = optimal specificity; κ[.5] = optimal effiency; κ[1] = optimal sensitivity.

Younger women = aged 18–34 years; mid-aged women = aged 35–59 years; older women = aged 60 years and older.

^a^Analyses with this subset of women could not be conducted due to the small sample size

## Discussion

This is the first study to examine the utility of the WHODAS 2.0 across PTSD definitions and modalities. Regardless of the *DSM* definition (*DSM-IV* versus *DSM-5*) or the assessment modality (interview versus self-report) used, WHODAS 2.0 scores differed significantly between veterans with and without PTSD, with medium to large effects. These findings are consistent with a large body of research documenting that the association between PTSD and impairment is typically characterized by medium to large effects [17,33–34]. We also examined the effect of gender and age on WHODAS 2.0 scores. Consistent with past research [35], men and women did not differ on the WHODAS 2.0 total score or any of the subscale scores; however, as might be anticipated, younger veterans were significantly less impaired than both mid-aged and older veterans on the WHODAS 2.0 total score and four of the six subscale scores.

Signal detection analyses indicated that the optimally efficient cutoff score on the WHODAS 2.0 for separating veterans with and without PTSD-related impairment was 32. Our cutoff score of 32 differs substantially from the cutoff score of 40 found by Marx and his colleagues [7]. This is likely because Marx et al. used a small, disability-seeking sample, whereas we employed a much larger, more diverse sample of veterans. Further, whereas Marx et al. used the interview version of the WHODAS 2.0, we used the self-report version. Finally, unlike Marx et al., we calculated measures of test quality, rather than measures of test performance, to determine the optimal cutoff score. Our use of test quality measures ensures that our findings are not unduly influenced by chance.

In response to Konecky and colleagues’ [13] concern that the utility of the WHODAS 2.0 might be undercut by varying reliability across demographic groups, we also examined whether different cutoff scores would be appropriate for different demographic subgroups. Findings suggested that the cutoff score of 32 generally appeared to be appropriate across men and women, and across younger, mid-aged, and older veterans (the optimally efficient cutoff score for all groups was 31 or 32, with κ[.5]s ranging from .38–.49). However, the importance of considering group membership was evident when age was considered within the context of gender. Although the best cutoff score for men regardless of age group was 31–32, for women, this was not the case. For younger women, cutoff scores of 28–34 demonstrated the same psychometric properties, and for mid-aged women, 34 was the optimally efficient cutoff score. This means that, although a cutoff score of 32 will generally capture probable PTSD-related disability across age and gender, it may produce additional false negatives among mid-aged men, both false positives and false negatives among younger women, and additional false positives among mid-aged women.

Relatedly, in this study, we chose our cutoff score by identifying the WHODAS 2.0 score with the greatest optimal efficiency (κ[.5]). Optimally efficient cutoff scores are recommended for differential diagnosis, because they minimize diagnostic errors by maximizing the number of agreements between test and diagnosis [30]. However, there may be settings where optimally sensitive or specific cutoff scores may be better suited. Scores with the greatest optimal sensitivity (κ[1]) are more lenient, identifying more individuals as having positive tests (i.e., they produce more false positives). For this reason, optimally sensitive cutoff scores are ideal for screening [30]. In contrast, cutoff scores with the greatest optimal specificity (κ[0]) are more stringent, and are ideal for confirming a diagnosis (i.e., they produce more false negatives) [30]. In our analyses, choosing an optimally efficient versus an optimally sensitive cutoff score would generally not change the recommended cutoff score. This was also the case for an optimally specific cutoff score across women and older men. However, if an optimally specific (rather than optimally efficient or sensitive) cutoff score was chosen for the total score and for younger and mid-aged men, the cutoff score would be higher. Similar to considerations discussed previously, this decision further highlights the importance of considering the purpose of the cutoff score to guide usage. The differences in cutoff scores across both certain demographic groups and optimally efficient, sensitive, and specific scores highlight the importance of considering context when choosing a cutoff score.

Taken together, our findings begin to answer APA’s call for additional research on the WHODAS 2.0 regarding its clinical utility. Our results suggest that the WHODAS 2.0 does show appropriate sensitivity to PTSD diagnostic status, indicating that it is likely capturing the impairment associated with the disorder. Further, although additional work is needed, the establishment of a cutoff score is the first step towards allowing clinicians and researchers to determine if different interventions cause either reliable or clinically significant change in PTSD-related impairment. Finally, our finding that cutoff scores may differ importantly across demographic subgroups highlights the necessity of a precision-based medicine approach; use of the WHODAS 2.0 may help clinicians and researchers better conceptualize how different groups benefit from different interventions, above and beyond measures of symptom severity.

The current findings also have important clinical implications. The development of a cutoff score that is representative of interviewer-rated PTSD-related disability is essential for establishing clinically significant impairment when the use of a clinical interview is not feasible (e.g., during disability compensation examinations). Importantly, the specification in the PTSD diagnosis requiring that symptoms cause clinically significant distress or impairment was intended to set the threshold for diagnosing the disorder and therefore avoid overpathologizing individuals who are not bothered or impaired by symptoms [3]. Without a cutoff score by which to operationalize this impairment, this specification cannot be assessed. Our findings will therefore allow disability examiners to use the WHODAS 2.0 in concert with a measure of PTSD symptom severity (e.g., the PCL) to establish whether individuals have clinically significant PTSD-related disability in addition to PTSD symptom levels consistent with the PTSD diagnosis. Having an established cutoff score will also be beneficial in clinical settings; it will allow for the identification of individuals with subthreshold PTSD who are experiencing clinically significant impairment so that they can be referred for appropriate care.

Strengths of the study include the use of a large sample collected across several VA medical centers that oversampled for women. Further, generalizability was increased by employing both self-report and clinical interview measures that assessed both *DSM-IV* and *DSM-5* PTSD criteria. Nonetheless, the study is not without limitations. First, due to the absence of an existing stand-alone interview designed to assess for PTSD-related impairment, we used two impairment items on the CAPS that were not created for this purpose. Second, although we oversampled for women, our sample of women (particularly older women) was quite small. This small sample size prohibited us from determining a cutoff score for older women. Relatedly, the κ[.5]s for women were consistently weaker than those for men; whereas [.5]s for women fell in the “poor” range, for men, they were in the “fair” range. These lower kappas among women may be due to the smaller sample size. Consistent with this, the largest group of women (mid-aged) had the highest kappas. Therefore, replication with a larger sample of women is needed. Finally, our sample included veterans who were seeking care at a VA. Thus, the degree to which the present findings generalize to other veteran or civilian populations is unknown.

Despite these limitations, this study provides additional evidence that the WHODAS 2.0 can be used to differentiate veterans with and without PTSD-related impairment. By establishing a cutoff score of 32, the study provides a threshold for interpreting WHODAS 2.0 scores in relation to the PTSD criterion of clinically significant impairment. Although this cutoff score can generally be used across groups, more research is needed to ensure appropriate cutoff scores for different groups, particularly women.
